# Crystal structure of 3-amino-5,5-dimethyl-2-[(*E*)-2-nitro­ethen­yl]cyclo­hex-2-en-1-one

**DOI:** 10.1107/S1600536814023009

**Published:** 2014-10-24

**Authors:** Brigita Vigante, Dmitrijs Stepanovs, Andrejs Pelss, Anatoly Mishnev

**Affiliations:** a Latvian Institute of Organic Synthesis, 21 Aizkraukles Street, Riga, LV-1006, Latvia

**Keywords:** nitro­dienamines, nitro­acetaldehyde, Knoevenagel-type condensation, crystal structure, hydrogen bonds

## Abstract

The asymmetric unit of the title compound, C_10_H_14_N_2_O_3_, contains two independent mol­ecules with similar conformations. In the both mol­ecules, the cyclo­hexene rings adopt the same envelope conformation with the flap C atoms lying 0.658 (3) and 0.668 (3) Å from the mean planes formed by the remaining atoms. In the crystal, adjacent mol­ecules are connected *via* N—H⋯O hydrogen bonds and weak C—H⋯O inter­actions, forming supra­molecular layers parallel to (-101).

## Abstract 2

The asymmetric unit of the title compound contains two independent mol­ecules with similar conformations, the cyclo­hexene rings adopting the same envelope conformation. In the crystal, adjacent mol­ecules are connected *via* N—H⋯O hydrogen bonds and weak C—H⋯O inter­actions, forming supra­molecular layers parallel to (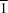
01).

## Chemical context   

*sec*-Nitro­dienamines appear to be potentially useful synthons in organic synthesis due to the enaminic, dienic and ‘push–pull’ character of these mol­ecules (Koike *et al.*, 2000 ▶). Several methods are available for the synthesis of nitro­dienamines, which include the reaction of acetaldehydes with 1-di­methyl­amino-2-nitro­ethylen followed by treatment with amines (Severin *et al.*, 1971 ▶), the reaction of amino­acrolein with di­methyl­amine and subsequent treatment of the vinyl­amidinium salt with nitro­methane (Takeuchi *et al.*, 1988 ▶) and nitro­alken­ylation reactions of 2-methyl­indolines with nitro­enam­ines (Attanasi *et al.*, 2006 ▶).
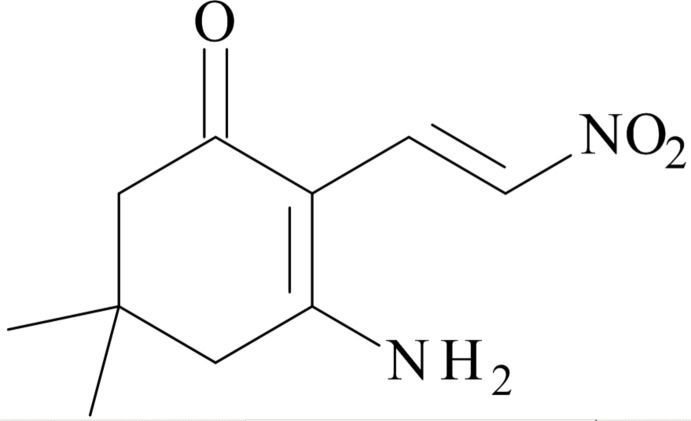


Previously, we found that alpha-nitro acetaldehyde undergoes an unusual condensation with aldehydes and ammonium acetate to afford 3,5-di­nitro-1,2-di­hydro­pyridines (Vigante *et al.*, 1993 ▶). Afterwards, the synthesis of *N*-substituted 1,2-di­hydro­pyridines by heterocyclic annulation reaction of *sec*-nitro­dienamines with acetaldehyde was reported (Koike *et al.*, 1999 ▶). As part of our studies of synthetic pathways to fused 1,2-di­hydro­pyridines, the title compound was synthesized and we report herein on its mol­ecular and crystal structure.

## Structural commentary   

**Figure 1:**
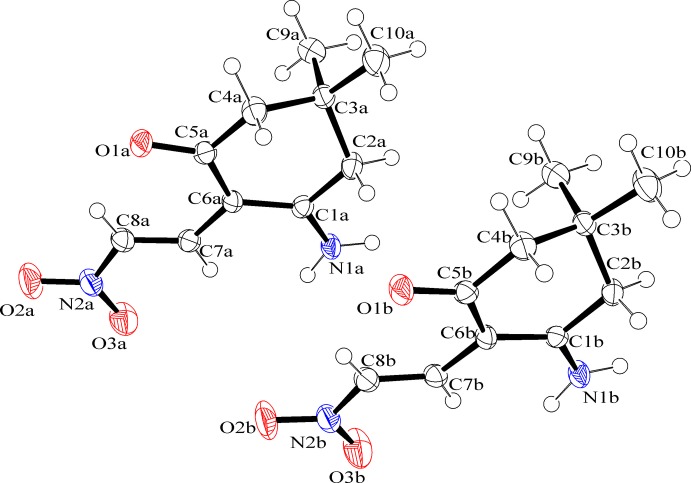
The asymmetric unit of the title compound, showing 50% probability displacement ellipsoids and the atomic numbering

The asymmetric unit of the title compound (Fig. 1 ▶) contains two independent mol­ecules (*A* and *B*) having coincident geometry. The bond lengths in the mol­ecules are close to standard values. The cyclo­hexene rings adopt an envelope conformation, with flap atoms C3*A* and C3*B* lying 0.658 (3) and 0.668 (3) Å from the mean planes formed by the remaining atoms in mol­ecules *A* and *B*, respectively.

## Supra­molecular features   

**Figure 2:**
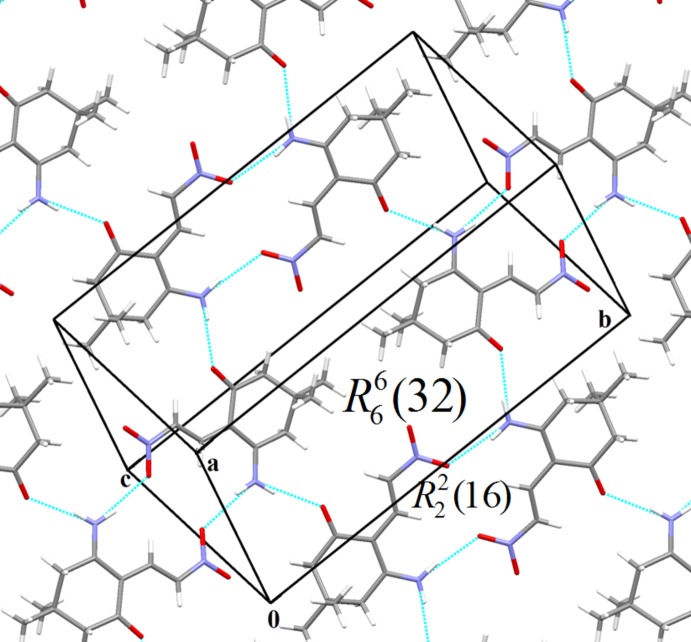
The crystal packing of the title compound showing sheets parallel to (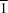
01).

In the crystal, the mol­ecules form sheets parallel to (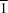
01) by means of N—H⋯O hydrogen bonds. The network consists of two hydrogen-bond motifs, 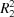
(16) and 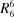
(32) (Fig. 2 ▶). Weak C—H⋯O inter­actions are also observed in the supra­molecular networks (Table 1 ▶).

## Database survey   

A search of the Cambridge Structural Database (Version 5.35; Groom & Allen, 2014 ▶) for 5,5-di­methyl­cyclo­hex-2-enones gave 609 hits. Only one of these is a 3-amino-5,5-di­methyl­cyclo­hex-2-enone, namely, 3-amino-5,5-dimethyl-2-phenyl­cyclo­hex-2-enone (Fun *et al.*, 2007 ▶). The conformation of the cyclo­hexene ring is identical to that found in the title compound.

## Synthesis and crystallization   

A mixture of 3-amino-5,5-di­methyl­cyclo­hex-2-enone (140 mg, 1 mmol) and potassium salt of alpha-nitro acetaldehyde (190 mg, 1.5 mmol) in methanol (2 mL) and acetic acid (2 mL) was stirred for 5 days at room temperature. The solvents were removed under reduced pressure and the residue was purified by flash chromatography on silica gel, eluent: chloro­form, hexane, acetone, methanol (9:7:1:1). The appropriate fraction was collected and crystallized from methanol, yielding 116 mg (55%) of bright-yellow crystals (m.p. 503 K).

MS (+ESI) *m*/*z* (relative intensity): 211.2 ([*M*+H]^+^, 100).^1^H NMR (400 MHz, DMSO-*d*_6_): *δ* 0.96 (*s*, 6H), 2.20 (*s*, 2H), 2.53 (*s*, 2H), 8.12 (*d*, *J* = 12.4 Hz, 1H), 8.39 (*d*, *J* = 12.4 Hz, 1H), 8.48 (*s*, 1H), 8.74 (*s*, 1H).

^13^C NMR (100.56 MHz, DMSO-d_6_): *δ* 27.94, 31.41, 44.12, 51.46, 100.02, 131.82, 132.15, 172.30, 193.93. Analysis calculated for C_10_H_14_N_2_O_3_: C, 57.13; H, 6.71; N, 13.32; found: C, 56.98; H, 6.78; N, 13.16.

## Refinement   

Hydrogens on the amino group were located in a difference Fourier map and freely refined. The C-bound hydrogen atoms were positioned geometrically with C—H distances ranging from 0.93 to 0.97 Å and refined as riding on their parent atoms with *U*_iso_(H) = 1.5*U*_eq_(C) for methyl groups and *U*_iso_(H) = 1.2*U*_eq_(C) for other H atoms. The reflection whose intensity was affected by the beamstop was removed from the final refinement. Crystal data, data collection and structure refinement details are summarized in Table 2 ▶.

## Supplementary Material

Crystal structure: contains datablock(s) I, globe. DOI: 10.1107/S1600536814023009/xu5825sup1.cif

Structure factors: contains datablock(s) I. DOI: 10.1107/S1600536814023009/xu5825Isup2.hkl

Supporting information file. DOI: 10.1107/S1600536814023009/xu5825Isup3.cml

CCDC reference: 1027341

Additional supporting information:  crystallographic information; 3D view; checkCIF report

## Figures and Tables

**Table 1:** Hydrogen-bond geometry (, )

*D*H*A*	*D*H	H*A*	*D* *A*	*D*H*A*
N1*A*H1*NA*O1*B* ^i^	0.84(2)	2.06(3)	2.873(2)	162(2)
N1*A*H2*NA*O3*B* ^ii^	0.88(2)	2.10(3)	2.961(2)	167(2)
N1*B*H1*NB*O3*A* ^ii^	0.92(2)	2.03(3)	2.942(2)	168(2)
N1*B*H2*NB*O1*A* ^iii^	0.85(3)	2.04(2)	2.858(2)	162(2)
C2*A*H2*A*1O1*B* ^i^	0.97	2.42	3.262(2)	145
C2*B*H2*B*1O1*A* ^iii^	0.97	2.46	3.298(2)	144
C7*A*H7*A*O3*B* ^ii^	0.93	2.54	3.469(2)	173
C7*B*H7*B*O3*A* ^ii^	0.93	2.55	3.469(2)	171

Symmetry codes: (i) 
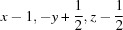
; (ii) 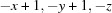; (iii) 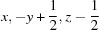.

**Table 2:** Experimental details

Crystal data	
Chemical formula	C_10_H_14_N_2_O_3_
*M*_r_	210.23
Crystal system, space group	Monoclinic, *P*2_1_/*c*
Temperature (K)	173
*a*, *b*, *c* ()	11.3545(3), 18.1097(5), 10.4689(3)
()	100.119(2)
*V* (^3^)	2119.20(10)
*Z*	8
Radiation type	Mo *K*
(mm^1^)	0.10
Crystal size (mm)	0.35 0.25 0.01

Data collection	
Diffractometer	Nonius KappaCCD
No. of measured, independent and observed [*I* > 2(*I*)] reflections	10741, 6174, 3235
*R*_int_	0.068
(sin /)_max_ (^1^)	0.705

Refinement	
*R*[*F* ^2^ > 2(*F* ^2^)], *wR*(*F* ^2^), *S*	0.069, 0.158, 1.01
No. of reflections	6174
No. of parameters	287
H-atom treatment	H atoms treated by a mixture of independent and constrained refinement
_max_, _min_ (e ^3^)	0.28, 0.29

Computer programs: *KappaCCD Server Software* (Nonius, 1997 ▶), *HKL* *DENZO* *SCALEPACK* (Otwinowski Minor, 1997 ▶), *SIR2011* (Burla *et al.*, 2012 ▶), *ORTEP-3 for Windows* (Farrugia, 2012 ▶), *SHELXL97* (Sheldrick, 2008 ▶), *PLATON* (Spek, 2009 ▶) and *publCIF* (Westrip, 2010 ▶).

## supplementary crystallographic information

### Crystal data

**Table:**

C_10_H_14_N_2_O_3_	*F*(000) = 896
*M**_r_* = 210.23	*D*_x_ = 1.318 Mg m^−^^3^
Monoclinic, *P*2_1_/*c*	Melting point: 503 K
Hall symbol: -P 2ybc	Mo *K*α radiation, λ = 0.71073 Å
*a* = 11.3545 (3) Å	Cell parameters from 11852 reflections
*b* = 18.1097 (5) Å	θ = 1.0–30.0°
*c* = 10.4689 (3) Å	µ = 0.10 mm^−^^1^
β = 100.119 (2)°	*T* = 173 K
*V* = 2119.20 (10) Å^3^	Plate, yellow
*Z* = 8	0.35 × 0.25 × 0.01 mm

### Data collection

**Table:**

Nonius KappaCCD diffractometer	3235 reflections with *I* > 2σ(*I*)
Radiation source: fine-focus sealed tube	*R*_int_ = 0.068
Graphite monochromator	θ_max_ = 30.1°, θ_min_ = 2.7°
CCD scans	*h* = −15→15
10741 measured reflections	*k* = −25→23
6174 independent reflections	*l* = −14→14

### Refinement

**Table:**

Refinement on *F*^2^	Primary atom site location: structure-invariant direct methods
Least-squares matrix: full	Secondary atom site location: difference Fourier map
*R*[*F*^2^ > 2σ(*F*^2^)] = 0.069	Hydrogen site location: inferred from neighbouring sites
*wR*(*F*^2^) = 0.158	H atoms treated by a mixture of independent and constrained refinement
*S* = 1.01	*w* = 1/[σ^2^(*F*_o_^2^) + (0.0554*P*)^2^ + 0.6139*P*] where *P* = (*F*_o_^2^ + 2*F*_c_^2^)/3
6174 reflections	(Δ/σ)_max_ < 0.001
287 parameters	Δρ_max_ = 0.28 e Å^−^^3^
0 restraints	Δρ_min_ = −0.29 e Å^−^^3^

### Special details

**Table:**

Geometry. All esds (except the esd in the dihedral angle between two l.s. planes) are estimated using the full covariance matrix. The cell esds are taken into account individually in the estimation of esds in distances, angles and torsion angles; correlations between esds in cell parameters are only used when they are defined by crystal symmetry. An approximate (isotropic) treatment of cell esds is used for estimating esds involving l.s. planes.
Refinement. Refinement of F^2^ against ALL reflections. The weighted R-factor wR and goodness of fit S are based on F^2^, conventional R-factors R are based on F, with F set to zero for negative F^2^. The threshold expression of F^2^ > 2sigma(F^2^) is used only for calculating R-factors(gt) etc. and is not relevant to the choice of reflections for refinement. R-factors based on F^2^ are statistically about twice as large as those based on F, and R- factors based on ALL data will be even larger.

### Fractional atomic coordinates and isotropic or equivalent isotropic displacement parameters (Å^2^)

**Table:**

	*x*	*y*	*z*	*U*_iso_*/*U*_eq_	
O1A	0.44423 (12)	0.26776 (8)	0.25500 (13)	0.0295 (4)	
O2A	0.55773 (14)	0.50926 (9)	0.20293 (17)	0.0471 (5)	
O3A	0.38755 (15)	0.52829 (9)	0.07970 (17)	0.0470 (5)	
N1A	0.09527 (15)	0.33969 (10)	−0.03942 (16)	0.0254 (4)	
H1NA	0.034 (2)	0.3223 (13)	−0.087 (2)	0.030*	
H2NA	0.1126 (19)	0.3869 (14)	−0.043 (2)	0.030*	
N2A	0.45967 (16)	0.48703 (10)	0.14688 (17)	0.0307 (4)	
C1A	0.16406 (16)	0.29331 (11)	0.03826 (18)	0.0209 (4)	
C2A	0.11184 (17)	0.21747 (11)	0.0458 (2)	0.0247 (5)	
H2A1	0.0619	0.2062	−0.0369	0.030*	
H2A2	0.0608	0.2179	0.1110	0.030*	
C3A	0.20444 (16)	0.15635 (11)	0.07868 (19)	0.0229 (4)	
C4A	0.28813 (18)	0.18035 (11)	0.20157 (19)	0.0271 (5)	
H4A1	0.2440	0.1807	0.2729	0.032*	
H4A2	0.3518	0.1442	0.2217	0.032*	
C5A	0.34312 (16)	0.25560 (11)	0.19190 (17)	0.0215 (4)	
C6A	0.27637 (16)	0.31205 (11)	0.11171 (17)	0.0207 (4)	
C7A	0.32346 (17)	0.38535 (11)	0.10523 (18)	0.0232 (4)	
H7A	0.2738	0.4188	0.0543	0.028*	
C8A	0.42972 (18)	0.41153 (11)	0.16320 (19)	0.0259 (5)	
H8A	0.4840	0.3805	0.2141	0.031*	
C9A	0.27396 (19)	0.14516 (13)	−0.0321 (2)	0.0329 (5)	
H9A1	0.3107	0.1909	−0.0500	0.049*	
H9A2	0.2202	0.1292	−0.1083	0.049*	
H9A3	0.3347	0.1084	−0.0078	0.049*	
C10A	0.14236 (19)	0.08427 (12)	0.1036 (2)	0.0356 (6)	
H10A	0.2015	0.0475	0.1344	0.053*	
H10B	0.0938	0.0676	0.0244	0.053*	
H10C	0.0927	0.0924	0.1677	0.053*	
O1B	0.92300 (13)	0.24275 (8)	0.28241 (13)	0.0322 (4)	
O2B	1.04280 (14)	0.48418 (9)	0.22269 (17)	0.0487 (5)	
O3B	0.88174 (16)	0.49987 (9)	0.08349 (18)	0.0521 (5)	
N1B	0.59376 (15)	0.31138 (10)	−0.04246 (17)	0.0263 (4)	
H1NB	0.6104 (19)	0.3611 (14)	−0.046 (2)	0.032*	
H2NB	0.537 (2)	0.2930 (12)	−0.097 (2)	0.032*	
N2B	0.94809 (15)	0.46084 (10)	0.16108 (17)	0.0315 (4)	
C1B	0.66003 (16)	0.26509 (11)	0.03703 (18)	0.0208 (4)	
C2B	0.61249 (16)	0.18770 (11)	0.03415 (19)	0.0233 (4)	
H2B1	0.5699	0.1773	−0.0526	0.028*	
H2B2	0.5553	0.1847	0.0927	0.028*	
C3B	0.70757 (16)	0.12831 (11)	0.07177 (19)	0.0227 (4)	
C4B	0.77921 (18)	0.15161 (11)	0.20286 (19)	0.0258 (5)	
H4B1	0.7281	0.1477	0.2677	0.031*	
H4B2	0.8451	0.1174	0.2267	0.031*	
C5B	0.82867 (17)	0.22863 (11)	0.20607 (18)	0.0224 (4)	
C6B	0.76642 (16)	0.28498 (11)	0.12114 (18)	0.0213 (4)	
C7B	0.81399 (17)	0.35818 (11)	0.11657 (18)	0.0236 (4)	
H7B	0.7678	0.3906	0.0596	0.028*	
C8B	0.91564 (18)	0.38621 (11)	0.18294 (19)	0.0258 (5)	
H8B	0.9651	0.3572	0.2432	0.031*	
C9B	0.78776 (19)	0.12275 (13)	−0.0302 (2)	0.0341 (5)	
H9B1	0.8237	0.1699	−0.0400	0.051*	
H9B2	0.7408	0.1080	−0.1116	0.051*	
H9B3	0.8493	0.0868	−0.0033	0.051*	
C10B	0.6467 (2)	0.05431 (12)	0.0841 (2)	0.0357 (6)	
H10D	0.7056	0.0184	0.1199	0.054*	
H10E	0.6074	0.0383	0.0000	0.054*	
H10F	0.5888	0.0596	0.1402	0.054*	

### Atomic displacement parameters (Å^2^)

**Table:**

	*U*^11^	*U*^22^	*U*^33^	*U*^12^	*U*^13^	*U*^23^
O1A	0.0257 (8)	0.0261 (9)	0.0313 (8)	0.0026 (6)	−0.0102 (6)	0.0006 (6)
O2A	0.0386 (9)	0.0392 (11)	0.0589 (11)	−0.0172 (8)	−0.0045 (8)	−0.0057 (8)
O3A	0.0501 (10)	0.0252 (9)	0.0583 (11)	−0.0029 (7)	−0.0110 (9)	0.0062 (8)
N1A	0.0226 (9)	0.0198 (10)	0.0294 (9)	−0.0021 (7)	−0.0073 (7)	0.0016 (7)
N2A	0.0321 (10)	0.0267 (11)	0.0314 (10)	−0.0052 (8)	0.0002 (8)	−0.0035 (8)
C1A	0.0194 (9)	0.0242 (12)	0.0187 (9)	0.0018 (8)	0.0021 (8)	−0.0004 (8)
C2A	0.0196 (10)	0.0243 (12)	0.0280 (10)	−0.0023 (8)	−0.0020 (8)	0.0045 (8)
C3A	0.0217 (10)	0.0193 (11)	0.0258 (10)	−0.0003 (8)	−0.0009 (8)	0.0010 (8)
C4A	0.0284 (11)	0.0238 (12)	0.0258 (10)	−0.0005 (9)	−0.0039 (9)	0.0059 (8)
C5A	0.0226 (10)	0.0244 (11)	0.0165 (9)	0.0035 (8)	0.0007 (8)	−0.0021 (8)
C6A	0.0206 (9)	0.0195 (11)	0.0201 (9)	0.0007 (8)	−0.0012 (8)	−0.0009 (7)
C7A	0.0248 (10)	0.0226 (12)	0.0207 (10)	0.0033 (8)	−0.0006 (8)	−0.0005 (8)
C8A	0.0275 (10)	0.0198 (12)	0.0286 (11)	−0.0003 (8)	0.0000 (9)	0.0000 (8)
C9A	0.0339 (11)	0.0270 (13)	0.0376 (12)	−0.0004 (9)	0.0061 (10)	−0.0051 (10)
C10A	0.0330 (12)	0.0248 (13)	0.0458 (14)	−0.0049 (9)	−0.0016 (11)	0.0055 (10)
O1B	0.0286 (8)	0.0280 (9)	0.0332 (8)	0.0016 (6)	−0.0131 (7)	−0.0021 (6)
O2B	0.0387 (9)	0.0368 (11)	0.0635 (12)	−0.0169 (8)	−0.0108 (9)	−0.0058 (8)
O3B	0.0520 (11)	0.0271 (10)	0.0665 (12)	−0.0061 (8)	−0.0193 (9)	0.0124 (8)
N1B	0.0229 (9)	0.0220 (10)	0.0292 (9)	−0.0008 (7)	−0.0090 (7)	0.0009 (7)
N2B	0.0302 (10)	0.0253 (11)	0.0361 (10)	−0.0038 (8)	−0.0018 (8)	−0.0039 (8)
C1B	0.0203 (10)	0.0211 (11)	0.0204 (9)	0.0023 (8)	0.0015 (8)	−0.0028 (8)
C2B	0.0189 (9)	0.0215 (11)	0.0276 (10)	−0.0024 (8)	−0.0013 (8)	0.0024 (8)
C3B	0.0216 (10)	0.0183 (11)	0.0269 (10)	−0.0013 (8)	0.0006 (8)	−0.0006 (8)
C4B	0.0274 (10)	0.0204 (11)	0.0271 (11)	0.0003 (8)	−0.0022 (9)	0.0051 (8)
C5B	0.0227 (10)	0.0226 (12)	0.0209 (10)	0.0027 (8)	0.0011 (8)	−0.0031 (8)
C6B	0.0203 (10)	0.0209 (11)	0.0207 (10)	−0.0003 (8)	−0.0017 (8)	−0.0010 (8)
C7B	0.0242 (10)	0.0206 (12)	0.0242 (10)	0.0024 (8)	−0.0007 (8)	−0.0019 (8)
C8B	0.0274 (10)	0.0230 (12)	0.0257 (10)	0.0013 (8)	0.0007 (9)	−0.0022 (8)
C9B	0.0329 (12)	0.0297 (14)	0.0405 (13)	−0.0033 (9)	0.0091 (10)	−0.0092 (10)
C10B	0.0378 (12)	0.0243 (13)	0.0419 (13)	−0.0072 (10)	−0.0017 (11)	0.0036 (10)

### Geometric parameters (Å, º)

**Table:**

O1A—C5A	1.240 (2)	O1B—C5B	1.245 (2)
O2A—N2A	1.231 (2)	O2B—N2B	1.228 (2)
O3A—N2A	1.233 (2)	O3B—N2B	1.230 (2)
N1A—C1A	1.324 (2)	N1B—C1B	1.320 (2)
N1A—H1NA	0.84 (2)	N1B—H1NB	0.92 (2)
N1A—H2NA	0.88 (2)	N1B—H2NB	0.86 (2)
N2A—C8A	1.426 (3)	N2B—C8B	1.430 (3)
C1A—C6A	1.410 (2)	C1B—C6B	1.411 (2)
C1A—C2A	1.504 (3)	C1B—C2B	1.500 (3)
C2A—C3A	1.524 (3)	C2B—C3B	1.526 (3)
C2A—H2A1	0.9700	C2B—H2B1	0.9700
C2A—H2A2	0.9700	C2B—H2B2	0.9700
C3A—C4A	1.522 (3)	C3B—C9B	1.523 (3)
C3A—C9A	1.527 (3)	C3B—C10B	1.524 (3)
C3A—C10A	1.528 (3)	C3B—C4B	1.527 (3)
C4A—C5A	1.510 (3)	C4B—C5B	1.502 (3)
C4A—H4A1	0.9700	C4B—H4B1	0.9700
C4A—H4A2	0.9700	C4B—H4B2	0.9700
C5A—C6A	1.449 (3)	C5B—C6B	1.453 (3)
C6A—C7A	1.437 (3)	C6B—C7B	1.435 (3)
C7A—C8A	1.338 (3)	C7B—C8B	1.338 (3)
C7A—H7A	0.9300	C7B—H7B	0.9300
C8A—H8A	0.9300	C8B—H8B	0.9300
C9A—H9A1	0.9600	C9B—H9B1	0.9600
C9A—H9A2	0.9600	C9B—H9B2	0.9600
C9A—H9A3	0.9600	C9B—H9B3	0.9600
C10A—H10A	0.9600	C10B—H10D	0.9600
C10A—H10B	0.9600	C10B—H10E	0.9600
C10A—H10C	0.9600	C10B—H10F	0.9600
			
C1A—N1A—H1NA	117.6 (15)	C1B—N1B—H1NB	123.2 (12)
C1A—N1A—H2NA	122.0 (13)	C1B—N1B—H2NB	117.2 (15)
H1NA—N1A—H2NA	120 (2)	H1NB—N1B—H2NB	119.4 (19)
O2A—N2A—O3A	121.86 (19)	O2B—N2B—O3B	121.85 (19)
O2A—N2A—C8A	118.09 (17)	O2B—N2B—C8B	117.90 (17)
O3A—N2A—C8A	120.05 (16)	O3B—N2B—C8B	120.26 (16)
N1A—C1A—C6A	124.16 (19)	N1B—C1B—C6B	124.21 (19)
N1A—C1A—C2A	114.59 (16)	N1B—C1B—C2B	114.67 (16)
C6A—C1A—C2A	121.23 (16)	C6B—C1B—C2B	121.11 (16)
C1A—C2A—C3A	114.37 (16)	C1B—C2B—C3B	114.68 (15)
C1A—C2A—H2A1	108.7	C1B—C2B—H2B1	108.6
C3A—C2A—H2A1	108.7	C3B—C2B—H2B1	108.6
C1A—C2A—H2A2	108.7	C1B—C2B—H2B2	108.6
C3A—C2A—H2A2	108.7	C3B—C2B—H2B2	108.6
H2A1—C2A—H2A2	107.6	H2B1—C2B—H2B2	107.6
C4A—C3A—C2A	106.68 (16)	C9B—C3B—C10B	109.70 (18)
C4A—C3A—C9A	110.52 (16)	C9B—C3B—C2B	110.40 (17)
C2A—C3A—C9A	110.67 (17)	C10B—C3B—C2B	109.33 (16)
C4A—C3A—C10A	109.75 (16)	C9B—C3B—C4B	111.01 (16)
C2A—C3A—C10A	109.79 (16)	C10B—C3B—C4B	110.17 (17)
C9A—C3A—C10A	109.39 (18)	C2B—C3B—C4B	106.16 (16)
C5A—C4A—C3A	113.90 (16)	C5B—C4B—C3B	114.51 (16)
C5A—C4A—H4A1	108.8	C5B—C4B—H4B1	108.6
C3A—C4A—H4A1	108.8	C3B—C4B—H4B1	108.6
C5A—C4A—H4A2	108.8	C5B—C4B—H4B2	108.6
C3A—C4A—H4A2	108.8	C3B—C4B—H4B2	108.6
H4A1—C4A—H4A2	107.7	H4B1—C4B—H4B2	107.6
O1A—C5A—C6A	121.64 (18)	O1B—C5B—C6B	121.31 (18)
O1A—C5A—C4A	118.67 (16)	O1B—C5B—C4B	118.76 (17)
C6A—C5A—C4A	119.68 (16)	C6B—C5B—C4B	119.93 (16)
C1A—C6A—C7A	120.33 (17)	C1B—C6B—C7B	120.06 (17)
C1A—C6A—C5A	118.48 (17)	C1B—C6B—C5B	118.28 (17)
C7A—C6A—C5A	121.18 (16)	C7B—C6B—C5B	121.58 (16)
C8A—C7A—C6A	128.25 (18)	C8B—C7B—C6B	128.93 (18)
C8A—C7A—H7A	115.9	C8B—C7B—H7B	115.5
C6A—C7A—H7A	115.9	C6B—C7B—H7B	115.5
C7A—C8A—N2A	120.01 (18)	C7B—C8B—N2B	119.90 (18)
C7A—C8A—H8A	120.0	C7B—C8B—H8B	120.0
N2A—C8A—H8A	120.0	N2B—C8B—H8B	120.0
C3A—C9A—H9A1	109.5	C3B—C9B—H9B1	109.5
C3A—C9A—H9A2	109.5	C3B—C9B—H9B2	109.5
H9A1—C9A—H9A2	109.5	H9B1—C9B—H9B2	109.5
C3A—C9A—H9A3	109.5	C3B—C9B—H9B3	109.5
H9A1—C9A—H9A3	109.5	H9B1—C9B—H9B3	109.5
H9A2—C9A—H9A3	109.5	H9B2—C9B—H9B3	109.5
C3A—C10A—H10A	109.5	C3B—C10B—H10D	109.5
C3A—C10A—H10B	109.5	C3B—C10B—H10E	109.5
H10A—C10A—H10B	109.5	H10D—C10B—H10E	109.5
C3A—C10A—H10C	109.5	C3B—C10B—H10F	109.5
H10A—C10A—H10C	109.5	H10D—C10B—H10F	109.5
H10B—C10A—H10C	109.5	H10E—C10B—H10F	109.5
			
N1A—C1A—C2A—C3A	152.82 (18)	N1B—C1B—C2B—C3B	152.97 (18)
C6A—C1A—C2A—C3A	−28.5 (3)	C6B—C1B—C2B—C3B	−28.1 (3)
C1A—C2A—C3A—C4A	52.7 (2)	C1B—C2B—C3B—C9B	−67.4 (2)
C1A—C2A—C3A—C9A	−67.5 (2)	C1B—C2B—C3B—C10B	171.80 (17)
C1A—C2A—C3A—C10A	171.61 (17)	C1B—C2B—C3B—C4B	53.0 (2)
C2A—C3A—C4A—C5A	−54.4 (2)	C9B—C3B—C4B—C5B	66.5 (2)
C9A—C3A—C4A—C5A	66.0 (2)	C10B—C3B—C4B—C5B	−171.79 (18)
C10A—C3A—C4A—C5A	−173.26 (18)	C2B—C3B—C4B—C5B	−53.5 (2)
C3A—C4A—C5A—O1A	−149.19 (18)	C3B—C4B—C5B—O1B	−150.83 (19)
C3A—C4A—C5A—C6A	31.9 (3)	C3B—C4B—C5B—C6B	29.2 (3)
N1A—C1A—C6A—C7A	−0.1 (3)	N1B—C1B—C6B—C7B	−5.0 (3)
C2A—C1A—C6A—C7A	−178.63 (19)	C2B—C1B—C6B—C7B	176.23 (19)
N1A—C1A—C6A—C5A	−179.33 (19)	N1B—C1B—C6B—C5B	178.3 (2)
C2A—C1A—C6A—C5A	2.2 (3)	C2B—C1B—C6B—C5B	−0.5 (3)
O1A—C5A—C6A—C1A	177.29 (19)	O1B—C5B—C6B—C1B	179.96 (19)
C4A—C5A—C6A—C1A	−3.9 (3)	C4B—C5B—C6B—C1B	−0.1 (3)
O1A—C5A—C6A—C7A	−1.9 (3)	O1B—C5B—C6B—C7B	3.3 (3)
C4A—C5A—C6A—C7A	176.91 (19)	C4B—C5B—C6B—C7B	−176.77 (19)
C1A—C6A—C7A—C8A	−176.7 (2)	C1B—C6B—C7B—C8B	−176.3 (2)
C5A—C6A—C7A—C8A	2.5 (3)	C5B—C6B—C7B—C8B	0.3 (3)
C6A—C7A—C8A—N2A	−178.8 (2)	C6B—C7B—C8B—N2B	178.4 (2)
O2A—N2A—C8A—C7A	179.2 (2)	O2B—N2B—C8B—C7B	−179.1 (2)
O3A—N2A—C8A—C7A	0.2 (3)	O3B—N2B—C8B—C7B	1.5 (3)

### Hydrogen-bond geometry (Å, º)

**Table:**

*D*—H···*A*	*D*—H	H···*A*	*D*···*A*	*D*—H···*A*
N1*A*—H1*NA*···O1*B*^i^	0.84 (2)	2.06 (3)	2.873 (2)	162 (2)
N1*A*—H2*NA*···O3*B*^ii^	0.88 (2)	2.10 (3)	2.961 (2)	167 (2)
N1*B*—H1*NB*···O3*A*^ii^	0.92 (2)	2.03 (3)	2.942 (2)	168 (2)
N1*B*—H2*NB*···O1*A*^iii^	0.85 (3)	2.04 (2)	2.858 (2)	162 (2)
C2*A*—H2*A*1···O1*B*^i^	0.97	2.42	3.262 (2)	145
C2*B*—H2*B*1···O1*A*^iii^	0.97	2.46	3.298 (2)	144
C7*A*—H7*A*···O3*B*^ii^	0.93	2.54	3.469 (2)	173
C7*B*—H7*B*···O3*A*^ii^	0.93	2.55	3.469 (2)	171

Symmetry codes: (i) *x*−1, −*y*+1/2, *z*−1/2; (ii) −*x*+1, −*y*+1, −*z*; (iii) *x*, −*y*+1/2, *z*−1/2.
